# Editorial: Insights in cardiovascular endocrinology: 2023

**DOI:** 10.3389/fendo.2023.1266221

**Published:** 2023-08-04

**Authors:** Gaetano Santulli

**Affiliations:** ^1^Department of Medicine, Wilf Family Cardiovascular Research Institute, Fleischer Institute for Diabetes and Metabolism (FIDAM), Albert Einstein College of Medicine, New York, NY, United States; ^2^Department of Molecular Pharmacology, Einstein-Mount Sinai Diabetes Research Center (ES-DRC), Einstein Institute for Aging Research, Institute for Neuroimmunology and Inflammation (INI), Albert Einstein College of Medicine, New York, NY, United States

**Keywords:** cardiology, endocrinology, metabolism, cardiovascular endocrinology, heart disease, metabolic disorders, cardiovascular medicine, diabetes mellitus

The present Research Topic entitled “*Insights in cardiovascular endocrinology: 2023*” aims at collecting the most updated reports in the field of Cardiovascular Endocrinology.

Cardiovascular endocrinology is an interdisciplinary field (**Figure 1**) that explores the intricate relationship between the endocrine system and cardiovascular health, including various areas of research, like the cross-talk between the cardiovascular system and other organs and systems, the involvement of the heart in systemic disorders; the functional role of hormones and regulatory peptides, produced by (or acting on) the cardiovascular system (1). Hormones play a significant role in regulating various physiological processes within the cardiovascular system, influencing vascular tone, cardiac function, and metabolism (2–5).

**Figure 1:**
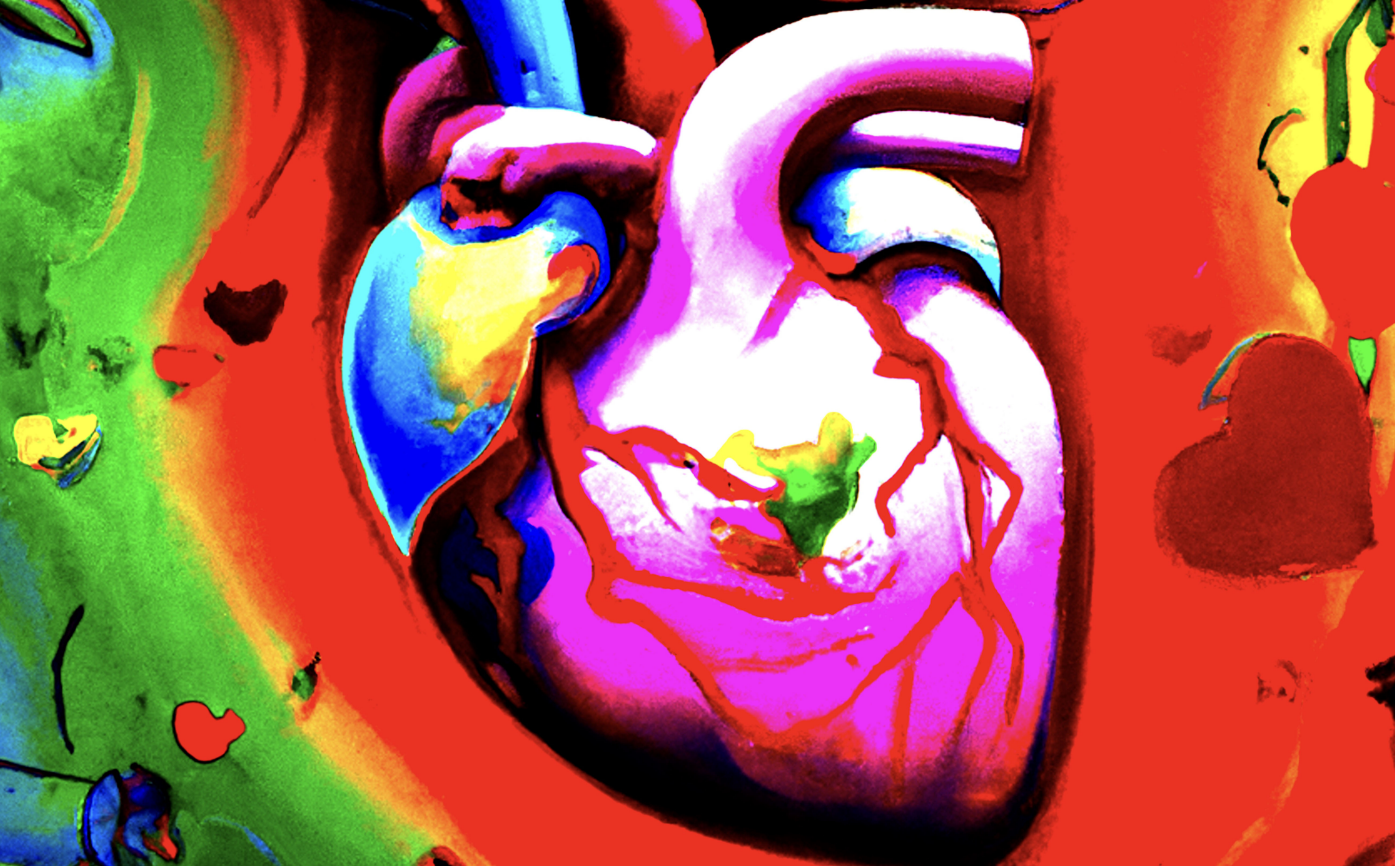
Artistic rendering of cardiovascular endocrinology.

Several hormones play crucial roles in cardiovascular regulation, including (but not limited to):

- Adrenaline and Noradrenaline: These catecholamines, produced by the adrenal medulla and sympathetic nerve endings, respectively, exert positive chronotropic and inotropic effects on the heart, increasing heart rate and contractility.
- Thyroid Hormones: Thyroid hormones, primarily thyroxine (T4) and triiodothyronine (T3), influence heart rate, cardiac contractility, and systemic vascular resistance; hypothyroidism and hyperthyroidism can lead to cardiovascular complications (6).
- Renin-Angiotensin-Aldosterone System (RAAS): The RAAS regulates blood pressure and fluid balance. Renin, secreted by the kidneys, converts angiotensinogen to angiotensin I, which is further converted to angiotensin II. Angiotensin II causes vasoconstriction and stimulates aldosterone release, leading to sodium and water retention (7).
- Insulin: Insulin affects vascular tone and glucose uptake in vascular smooth muscle cells. Insulin resistance and hyperinsulinemia have been linked to endothelial dysfunction and atherosclerosis.
- Natriuretic Peptides: Atrial natriuretic peptide (ANP) and brain natriuretic peptide (BNP) are released in response to increased cardiac wall stretch. They promote vasodilation and natriuresis, helping to reduce blood pressure and maintain fluid balance (8). Equally important, endocrine disorders can significantly impact cardiovascular health.

In the first article that opens this Research Topic, Dong and Yang authoritatively describe the trends in lipid profile and lipid control among survivors of stroke or myocardial infarction among US adults in ~ two decades (2001–2018), showing that lipid concentrations decreased and lipid control improved in stroke and/or myocardial infarction survivors, with heterogeneity observed according to sex and race. In an elegant review, the “CArdiometabolic Panel of International experts on Syndemic COvid-19” (CAPISCO) coordinated by Manfredi Rizzo reports on the key role of telemedicine for diabetes management during COVID-19, aiming at a future humanized digitalization (Rosta et al.). Next, Forzano et al. highlight how the new selective aldosterone synthase inhibitor Baxdrostat leads to significant reduction in both systolic and diastolic blood pressure in patients with resistant hypertension, representing a new powerful tool to treat resistant hypertension. In a comprehensive overview, Cheng et al. examine the central role of cardiac fibroblasts in myocardial fibrosis of diabetic cardiomyopathy. In the next review article, the favorable effects of choline supplements are examined in detail (Kansakar et al.). The last two articles, both written by the research group led by Esma Isenovic, neatly analyze the essential roles of insulin-like growth factor 1 (IGF-1) for maintaining cardiovascular health (Macvanin et al.) and the potential therapeutic opportunities offered by targeting the sodium/potassium adenosine-triphosphatase (Na^+^/K^+^-ATPase) in cardiometabolic disorders (Obradovic et al.).

In cardiovascular endocrinology, the diagnosis and management of endocrine disorders with cardiovascular implications are critical. The evaluation may include hormone assays, imaging studies, and functional tests to assess cardiovascular function. Treatment of cardiovascular endocrine disorders aims to restore hormonal balance and optimize cardiovascular health. Therapeutic interventions may include hormone replacement therapy, antihypertensive medications, lipid-lowering agents, and lifestyle modifications. Advancements in cardiovascular endocrinology are expected to provide insights into novel therapeutic targets and personalized treatment strategies for patients with cardiovascular diseases and endocrine disorders. Indeed, hormones play vital roles in regulating cardiovascular physiology, and disturbances in the endocrine system can have significant implications for cardiovascular function. Understanding these interactions is essential for diagnosing and managing endocrine disorders that impact cardiovascular health, ultimately leading to improved patient outcomes.

## Author contributions

GS: Conceptualization, Writing – original draft, Writing – review & editing.
